# An Optimal CDS Construction Algorithm with Activity Scheduling in Ad Hoc Networks

**DOI:** 10.1155/2015/842346

**Published:** 2015-05-27

**Authors:** Chakradhar Penumalli, Yogesh Palanichamy

**Affiliations:** Department of Information Science & Technology, Anna University, Chennai 600025, India

## Abstract

A new energy efficient optimal Connected Dominating Set (CDS) algorithm with activity scheduling for mobile ad hoc networks (MANETs) is proposed. This algorithm achieves energy efficiency by minimizing the Broadcast Storm Problem [BSP] and at the same time considering the node's remaining energy. The Connected Dominating Set is widely used as a virtual backbone or spine in mobile ad hoc networks [MANETs] or Wireless Sensor Networks [WSN]. The CDS of a graph representing a network has a significant impact on an efficient design of routing protocol in wireless networks. Here the CDS is a distributed algorithm with activity scheduling based on unit disk graph [UDG]. The node's mobility and residual energy (RE) are considered as parameters in the construction of stable optimal energy efficient CDS. The performance is evaluated at various node densities, various transmission ranges, and mobility rates. The theoretical analysis and simulation results of this algorithm are also presented which yield better results.

## 1. Introduction

Mobile ad hoc networks appear in a wide variety of applications such as the military, disaster relief, surveillance, sensing, and monitoring. Mobile ad hoc network (MANET) is a special kind of wireless network environment. It is different from the traditional wireless networks. Ad hoc network is a collection of autonomous and arbitrarily located wireless nodes. It is an infrastructure less network with dynamic topology, limited link bandwidth, variation in links, node capabilities, and energy-constrained resources. The nodes need to be more intelligent as they have to act as sender, receiver, and intermediate forwarder. Because of dynamic topology they frequently advertise the control information using a simple flooding. Simple flooding leads to Broadcast Storm Problem [1]. Broadcast Storm refers to the problem of exhausting the limited resources of the network due to excessive transmission of packets in the network. Considering this environment, several routing protocols have been proposed in order to find out and maintain the multihop route with reliability.

Generally ad hoc routing protocols in MANETs are classified into two types, namely, reactive and proactive. In proactive (table driven) routing protocols like DSDV [2] and OLSR [3] routing information in the network to update the tables is periodically broadcast. In case of on-demand (reactive) ad hoc routing protocols such as DSR [4] and AODV [5] simple flooding is used in route discovery procedure by broadcasting route request (RREQ) packets to the network. Generally in MANETs flooding is also used for* alarm signals, paging*, and so forth. However reactive routing protocols do not involve flooding of data packets but flooding of control packets takes place. Simple flooding consumes energy of the nodes and bandwidth of the network and also leads to congestion in the network which is called Broadcast Storm Problem.

In simple flooding a host on receiving a broadcast message for the first time will rebroadcast the message into network. A straightforward approach of simple flooding in spite of the fact that size of control packets is small suffers from the Broadcast Storm Problem which leads to the following problems [1].

1. *Redundant Rebroadcasts.* When a mobile host decides to rebroadcast a broadcast message to its neighbours, all its neighbours already have the message.
2. *Contention for Medium.* After a mobile host broadcasts a message, if many of its neighbours decide to rebroadcast the message, then they contend with each other.
3. *Collision.* Collisions are more likely to occur due to lack of efficient back-off mechanism.

The effect of the Broadcast Storm Problem can be reduced efficiently and effectively using Connected Dominating Set (CDS). A Connected Dominating Set is constructed assuming the MANET as a graph, generally unit disk graph [6] for better approximation. CDS is analogous to the backbone network of traditional wired networks. The activity of broadcasting is confined to the nodes of CDS. The smallest possible CDS is called Minimum Connected Dominating Set (MCDS) and the problem of constructing MCDS is an NP hard problem. Many standard methods exist in the literature to construct the Minimum Connected Dominating Set [7–23]. Each has its own merits and demerits. Activity scheduling can be used to prolong the lifetime of the network by rotating the role of various nodes [24]. In this paper we propose an energy efficient optimal CDS algorithm with activity scheduling. Scheduling between the similar neighbourhood backbone nodes (dominators) improves the energy efficiency at the same time. Here the activity scheduling is distributed and synchronous which suits much MANETs. It has also less message complexity because only the dominator nodes involve scheduling locally.

The rest of the paper is organized as follows: The definitions, mathematical calculations, and notations used in this paper are given in Section 2. The existing solutions are reviewed in Section 3. The proposed work is discussed in Section 4. The performance analysis and simulation results are shown in Section 5. The conclusion and future work are provided in Section 6.

## 2. Definitions, Mathematical Calculations, and Notations

Some important definitions, mathematical equations, and the notations used in this work are discussed here.

### 2.1. Definitions

*G* = (*V*, *E*) is a unit disk graph [6] which represents ad hoc wireless network. “*V*” is the set of mobile nodes in the network and “*E*” represents all the links in the network. We assume that all the nodes are deployed in a 2-D plane and their maximum transmission range is the same initially.

**Definition 1 .**

Any node in an UDG is adjacent to at most five independent nodes.

**Definition 2 .**

A dominator is a node which rebroadcasts the messages in the network. Generally it is called a BLACK node.

**Definition 3 .**

A Dominatee is node which listens to the broadcast message from dominator node. It will not rebroadcast the message. Usually it is called a GREY node.

**Definition 4 .**

A dominating set (DS) of a graph *G* = (*V*, *E*) is a subset of “*V*” such that each node in “*V*” is adjacent to at least one node in dominating set.

**Definition 5 .**

A Connected Dominating Set (CDS) “*C*” of *G* = (*V*, *E*) is a dominating set of *G* which induces a connected subgraph of *G*.

**Definition 6 .**

Minimum Connected Dominating Set (MCDS) is a CDS with minimum cardinality.

### 2.2. Mathematical Calculations for Energy

The energy calculations and equations used in this work are described here. All calculations are in joules. In the network, only the energy of dominator nodes is considered because they consume more energy when compared to the Dominatees. Here dominators consume more power than Dominatees because the set of dominators acts as the backbone. Moreover there are many power consuming roles performed by dominators. First a dominator has to rebroadcast the messages in its transmission range. Second it has to keep track of its connectors to the other dominators. Third it has to maintain a table for list of active neighbours. Fourth it has to perform local repairs. Finally it has to perform activity scheduling with its counterparts. All these indulge in high message exchange and in turn energy consumption.

Moreover the Dominatees are considered to be in promiscuous mode in the case of exchanging control packets alone where they consume very low power to receive the control messages. We considered only the transmission of control packets and not data packets. The Dominatees do not have much role in the construction of the CDS and hence throughout the construction phase Dominatees remain in promiscuous mode. The power consumption at various modes is shown in Table 3. Equations (4), (5), and (6) give a clear picture of how energy is consumed for each bit. When compared to the rate at which energy is depleted in the dominators, the rate at which energy is depleted in the Dominatees is negligible.

In order to increase the lifetime of the dominator as well as network we have performed the activity scheduling to balance the energy consumption. At the same time we can make the network fault tolerant to some extent.

The dominator node's energy “*E*_*d*_” is a function of the residual energy (RE) and dissipated energy (DE) as given in (1) and the energy dissipation rate (*D*) given in (2):

(1) $$\begin{array}{c} E_{d}=f\left(\;\text{RE},\text{DE}\;\right). \end{array}$$

The dissipation rate at a random time

(2) $$\begin{array}{c} D_{i}\left(\;t\;\right)=\frac{\left(E_{di\left(0\right)}-E_{di\left(t\right)}\right)}{t}, \end{array}$$

where *E*_*di*(0)_ = energy of node *i* at time *T* = 0 and *E*_*di*(*t*)_ = energy of node *i* at time *T* = *t*.

Let the initial energy of the dominator be “*E*_max_.” Once the dominator starts functioning, the energy is consumed and the available energy is reduced. The energy available at a particular instant of time is called residual energy (RE). Let “*s*” be the initial duration for which the dominator has functioned. After the time duration “*s*” the residual energy available at the dominator is computed as in

(3) $$\begin{array}{c} {\text{RE}}_{d}=E_{max}-{\text{DE}}_{s}, \end{array}$$

where “RE_*d*_” is the residual energy of the dominator and “DE_*s*_” is the energy dissipated during duration *s*.

The dissipated energy DE_*s*_ is calculated as follows.

Let “*B*_*t*_” be the energy required to transmit a bit and let “*B*_*r*_” be the energy required to receive a bit. Let “*B*_*p*_” be the energy consumed during the promiscuous mode of operation in one time unit. “*B*_*r*_” is nearly half of “*B*_*t*_” and “*B*_*p*_” is one-tenth of “*B*_*t*_.” Let “*m* ^1^” be the number of bits transmitted during “*s*” and let “*n* ^1^” be the number of bits received during “*s*.” The depleted energy during duration “*s*” is computed as in

(4) $$\begin{array}{c} {\text{DE}}_{s}=m^{1}*B_{t}+n^{1}*B_{r}+s*B_{p}. \end{array}$$

For the subsequent time intervals of duration “*s*” the available energy is computed as in

(5) EcurREd,REd=Ecur−DEs,

where *E*_cur_ is the residual energy available at a particular instant in time.

The overall remaining energy of the backbone network is computed as in

(6) $$\begin{array}{c} {\text{RE}}_{n}=\overset{n}{\underset{i=0}{\sum }}\left(\;E_{cur}-{\text{DE}}_{s}\;\right), \end{array}$$

where RE_*n*_ = The overall residual energy of the backbone network.

RE_*d*_ is calculated at regular intervals, if this ratio for the dominator becomes less than the threshold (TH) then it will choose another equivalent node and performs activity scheduling. Here in this algorithm we have put TH as 10% of *E*_max_ because of energy requirement. We have simulated the network with various values of threshold TH (5%, 10%, 15%, and 20%) of *E*_max_ and observed that when TH = 10% of *E*_max_ maintains the tradeoff between network lifetime and message cost. When TH = 20% of *E*_max_ then frequent disconnections occur which result in high message cost and low network lifetime. When TH = 5% of *E*_max_ then network lifetime increases but the dominator node will become unavailable because the node's energy will completely drain out. The results are shown in Figure 15. Here the network is simulated with 200 nodes at various transmission ranges.

So threshold is kept as 10% of *E*_max_.

That is,

(7) $$\begin{array}{c} \text{TH}=0.1\left(\;E_{max}\;\right). \end{array}$$

RE ratio is given by

(8) $$\begin{array}{c} \text{RE }\left(\;\text{ratio}\;\right)=\frac{\left(E_{max}-{\text{RE}}_{i-1}\right)}{E_{max}}. \end{array}$$

Generally, 0 < RE(ratio) < 1.

### 2.3. Notations

The notations used in this work are given in the Notations section:

 Δ : the degree of the node that has highest number of neighbours in the graph,
 
*H*: harmonic function,
 Big*O*: the Asymptotic upper bound of a function,
 
*Ω*: the Asymptotic lower bound of a function,
 
*θ*: tight bound, that is, both upper and lower bounds are of the same order,
 
*G*: unit disk graph,
 
*V*: number of vertices (nodes),
 
*E*: number of edges (links),
 
*E*_*d*_: energy of a dominator,
 
*B*_*t*_: transmission energy of dominator,
 
*B*_*r*_: receiving energy of dominator,
 
*B*_*p*_: energy required in promiscuous mode,
 
*N*_1_: 1-hop neighbors,
 
*N*_*i*1_: 1-hop neighbor list of node *i*, where *i* = 1,2,…, *n*,
 Δ*T*: random time interval,
 
*δT*: small time interval used for activity scheduling,
 Φ: null set (or) empty set,
 
*n*: number of nodes in the network,
 
*s*: time duration,
 RE_*d*_: the residual energy of the dominator,
 
*E*_cur_: current residual energy,
 DE_*s*_: the energy dissipated during duration “*s*,”
 |OPT|: the optimal value,
 |MCDS|: size of MCDS.

## 3. Literature Survey

### 3.1. Existing Solutions

There are various solutions to alleviate this Broadcast Storm Problem [1]. Some important solutions are probability based scheme, area based scheme, counter based scheme, neighbor knowledge based scheme, and CDS based scheme [1, 7–23, 25–27]. However except CDS based scheme none of these schemes are able to form the virtual backbone network. Connected Dominating Set (CDS) [7–23] belongs to graph based method. It can be used as a virtual backbone or spine of wireless ad hoc networks. CDS provides efficiency not only in broadcasting but also in multicasting and power management. Most of the above CDS algorithms aimed at small size of CDS but not considered the lifetime of CDS. Our algorithm considers both the size of CDS and lifetime of CDS. Generally they are classified into global, quasi-global, local, quasi-local ones based on the type of information it gathers. Generally the CDS construction algorithms are classified into two types: centralized and distributed.

#### 3.1.1. Centralized CDS Algorithms

Centralized algorithms require entire network topology (global) of the MANET. They produce small sized CDS and better performance ratio when compared to distributed CDS algorithms. But they are prone to the problems caused by the mobility of nodes. Guha and Khuller [10] propose two polynomial time algorithms to construct a CDS in a general graph *G*. These algorithms are greedy and centralized. The first one has an approximation ratio of 2(*H*(Δ) + 1), where “*H*” is a harmonic function and “Δ” is the degree of the node that has the highest number of neighbours in *G*. Here a spanning tree is developed with maximum degree node as root. The nonleaf nodes in the tree forms the CDS. The second algorithm constructs a Steiner tree that connects all dominator (BLACK) nodes to form CDS. The size of CDS is at most (ln(Δ) + 3) | OPT|, where |OPT| is the size of an optimal MCDS. The message complexity is *O*(*n* ^2^) and time complexity is also *O*(*n* ^2^). Alzoubi et al. [14] propose a one-step greedy approximation algorithm with performance ratio at most 3 + ln(Δ). Butenko et al. [16] propose a two-phase algorithm; in first phase Maximum Independent Set (MIS) is constructed and second phase forms the CDS based on Steiner tree with minimum number of Steiner nodes. It has a performance ratio of 6.8|MCDS|. Cheng et al. [11] proposed a three-phase algorithm (centralized) to construct MCDS in UDG. It has time complexity of *O*(*n*) and message complexity of *O*(*n*). Stojmenovic et al. [17] proposed a pruning based CDS construction. It has time complexity of *O*(|*V*|·|*E*|) and message complexity of *O*(*n* ^2^log^3^⁡*n*). In centralized solutions mobility is a concern. Due to mobility topology changes which reflect in the frequent update cost. In our work we follow a distributed approach for the construction of the CDS.

#### 3.1.2. Distributed CDS Algorithms

CDS is constructed based on localized information. These algorithms are further classified into prune based and MIS based ones. Wan et al. [8] proposed a distributed algorithm based on MIS this algorithm has time complexity of *O*(*n*) and message complexity of *O*(*n*log(*n*)). The resulting CDS has a size of at most 8 | OPT | + 1. Wu and Li's algorithm [7] is very simple. The localized property makes the CDS maintenance easier. The algorithm has a linear performance ratio. This algorithm needs at least two-hop neighbourhood information. It is presented based on the general graph model and it has time complexity of *θ*(*m*) and message complexity of *O*(Δ^3^). They analysed the movement of nodes with an on-model or off-model as leaving from one small management domain and entering another management domain. Das and Bharghavan [12] propose a three-staged algorithm; first stage identifies dominating set, second stage constructs spanning forest, and third stage constructs the spanning tree. It has a time complexity of *O*(*n* ^2^), message complexity of *O*(*n* ^2^), and cardinality is at most 3*H*(Δ). Stojmenovic et al. [17] propose a cluster based CDS construction which is based on 2-hop neighbourhood knowledge. Here ranking is based on degree and location of the node. It has time complexity of Ω(*m*) where “*m*” is number of edges and message complexity of *O*(*n* ^2^). Khabbazian et al. [22] proposed a local broadcast algorithm based on positional information to construct CDS. It has not focused much on mobility and energy. Sakai et al. [21] proposed a timer based CDS algorithm: two versions, namely, the single initiator CDS algorithm and multi-initiator CDS algorithm. Single initiator algorithm requires less time to construct the small size CDS when compared to multi-initiator. The message complexity for the multi-initiator is very high, but it has not concentrated much on the energy. Li et al. [18] proposed an algorithm with two phases. At the first phase, a Maximal Independent Set (MIS) is formed. At the second phase, a Steiner tree algorithm is used to connect the MIS. Cheng et al. [11] proposed a distributed multileader algorithm. It selects the node with minimum ID in its 1-hop neighbourhood as a leader, builds a tree rooted at each leader, and connect two adjacent trees through one or two nodes. It has a message complexity of *O*(*n*log*n*) and time complexity of *O*(*n*). Tang et al. [28] proposed an energy efficient MCDS algorithm which considered the energy before constructing the MCDS algorithm. It has not much concentrated on mobility and also it is based on reduced neighbour set. Here two stages are used one is CDS construction stage and the second is pruning the CDS to MCDS. It has not concentrated on mobility. Leu and Chang [20] proposed a distributed algorithm for Relaying Mobile Node (RMN) with neighbour table (*N*) and finally formed a CDS. Most of the above distributed algorithms concentrated either on mobility or on energy but not on both. The complexities of some important basic algorithms are detailed in Table 1.

### 3.2. Activity Scheduling

The lifetime of a CDS can be significantly increased by allowing some of the neighbouring dominators to sleep intermittently. Activity scheduling must fulfill two requirements: connectivity and coverage and minimization of the energy utilization. There are several sleep scheduling algorithms existing in the literature [24, 29]. These scheduling algorithms are classified into centralized and distributed ones. Distributed scheduling suits mobile ad hoc networks since MANETs lack infrastructure and centralized authority. As in the literature these activity scheduling schemes are further classified into scheduled wakeups and on-demand wakeups [30]. Miller and Vaidya [31] proposed a MAC layer activity scheduling algorithm that uses idle time to sense the channel. Sumi et al. [32] proposed an rotation based activity scheduling for wireless routing protocols where the roles of active and sleep are rotated among the MIS (maximal independent sets) based on power level priority. Kumar et al. [33] proposed a self-scheduling randomized independent scheduling (RIS) which uses synchronized probabilistic method which takes QOS as the metric. Berman et al. [34] proposed scheduling algorithm based on maximization problem to achieve *K*-coverage. In much of the above scheduling algorithms almost all nodes involve activity scheduling which result in high message complexity.

Here we have designed an activity scheduling that suits MANETs and also the scheduling happens between dominators only. At a time dominator node may be in active mode (WAKEUP) or promiscuous mode (SLEEP). The dominating node in promiscuous mode sniffs the channel for wakeup signal or sleep signal at regular intervals. Moreover, the proposed activity scheduling algorithm in this paper also considers energy of the dominators.

## 4. Proposed Work

The proposed energy efficient optimal CDS algorithm with activity scheduling is a distributed algorithm. All nodes are assumed to be of equal energy and equal transmission range initially. Our work considers both energy and mobility of the nodes in the construction of MCDS. In addition, we employ activity scheduling algorithm to spend the energy in a more economical way. The actual algorithm is divided into five phases. Phase 1 deals with dominator election. Phase 2 is about obtaining the connectors across the network and forming redundant CDS. Phases 3 and 4 deal with the construction of optimal CDS algorithm along with activity scheduling among dominators to increase the lifetime of dominators and in turn increase the lifetime of the network. Activity scheduling performs localized scheduling among different dominators with same set of *N*_1_ list so that the energy of the dominators sustain. Phase 5 is about the maintenance of the CDS at frequent intervals. Generally the CDS is disturbed when a dominator moves away or a new node joins the network or the energy and transmission range of the existing dominator goes below the threshold (TH) value. Here the residual energy and the transmission range of a dominator is calculated at regular intervals because initially all nodes have equal energy. In later states the nodes energy reduces according to (1), (2), (3), and (4).

*Algorithm Phase 1*. This phase is the initial phase, executed distributively over the network. Initially all nodes are in WHITE colour and have equal energy and transmission range. So, residual energy (RE) has no role in selecting the dominating set but in later stages of reconstruction/maintenance of CDS, RE plays an important role in selecting energy efficient CDS algorithm (see Algorithm 1).

**Figure 1:**
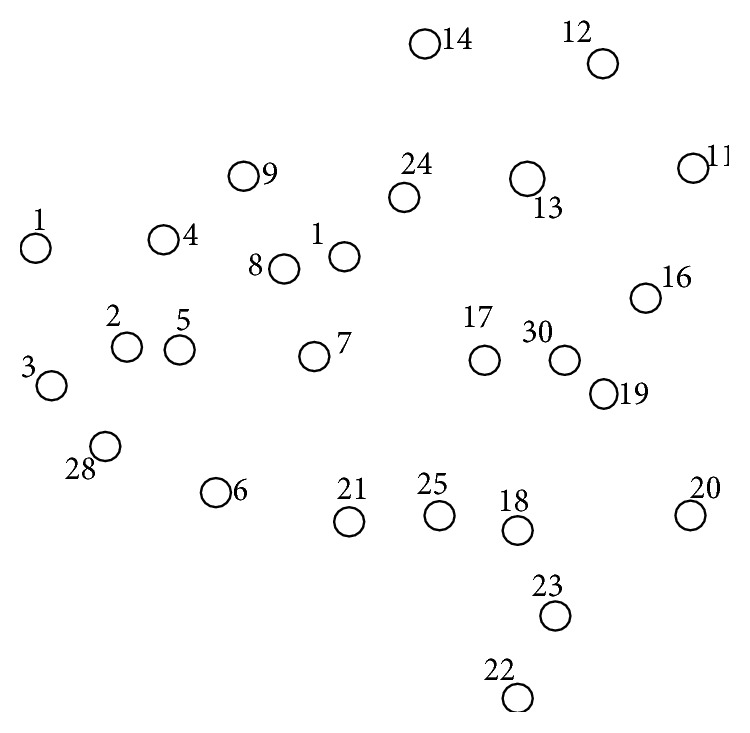
Initial example MANET.

**Figure 2:**
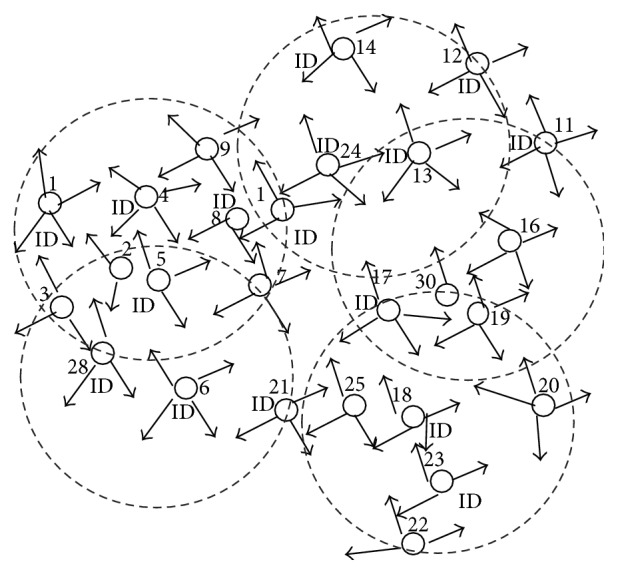
Initial MANET with ID exchange.

**Figure 3:**
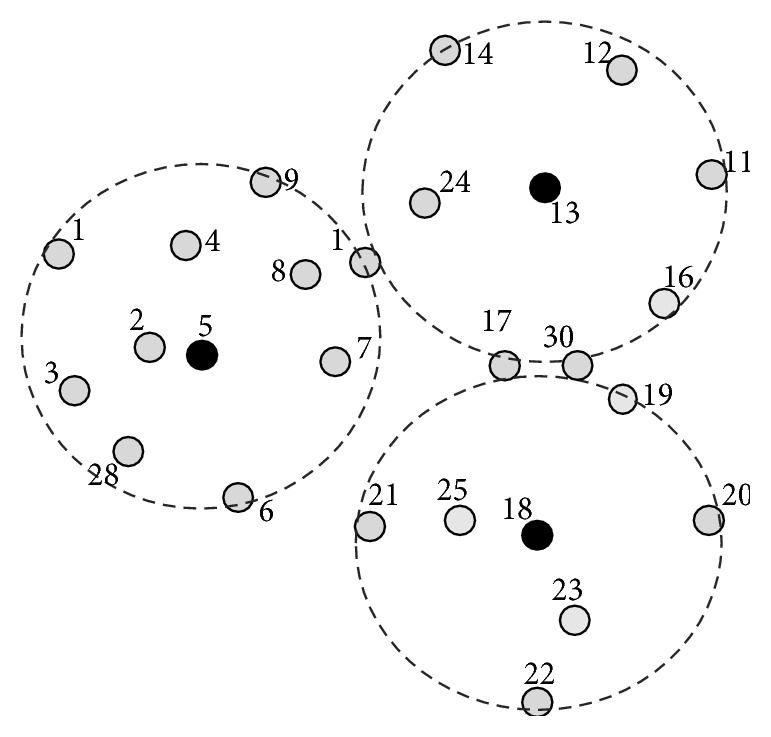
MANET after algorithm phase 1 with DS.

This phase is illustrated with sample MANET topology as shown in Figure 1. After the exchange of IDs and RE (initially not counted) as shown in Figure 2, the nodes with the highest number of neighbors and lowest IDs form as dominating set, DS (BLACK nodes) = {5,13,18} as shown in Figure 3. After this algorithm, phase 2 is implemented.

*Algorithm Phase 2*. This is the connection establishment phase. That is, connectors are selected to connect the DS and to form CDS (see Algorithm 2).

**Figure 4:**
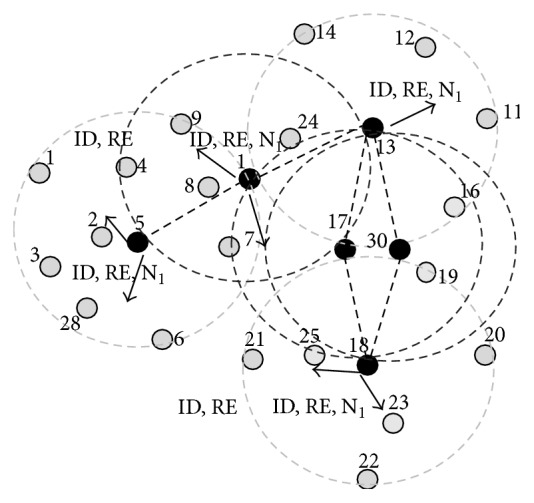
Connectors are selected after algorithm phase 2 and (ID, RE, and *N*_1_) are exchanged to get equivalent node for performing activity scheduling.

This phase is illustrated here in the example MANET; the GREY nodes with two different BLACK nodes are node 1, node 17, and node 30. Now nodes 1, 17, and 30 will turn to BLACK as shown in Figure 3 and connections are established. After this phase CDS is formed as shown in Figure 4. In the next phase equivalent counterparts are selected locally by these dominators for activity scheduling.

*Algorithm Phases 3 and 4*. This phase plays a crucial role where it prunes the previous CDS to obtain optimal CDS and it performs activity scheduling (Phase 4) among different adjacent dominators (BLACK nodes) with at most the same *N*_1_ list to conserve the dominator's energy. Activity scheduling shifts the role of dominators at intervals, *δT* and their residual energies RE_*s*_ are also calculated as shown in (3). That is, the equivalent adjacent dominators turn on (WAKEUP) and off (SLEEP) alternatively to conserve the energy and increase the lifetime without compromising the delivery ratio. Here a new regular interval based activity scheduling algorithm is designed to suit the mobility of nodes. Here we maintained a trade-off between optimal CDS and number of dominators to perform the activity scheduling (see Algorithms 3 and 4).

**Figure 5:**
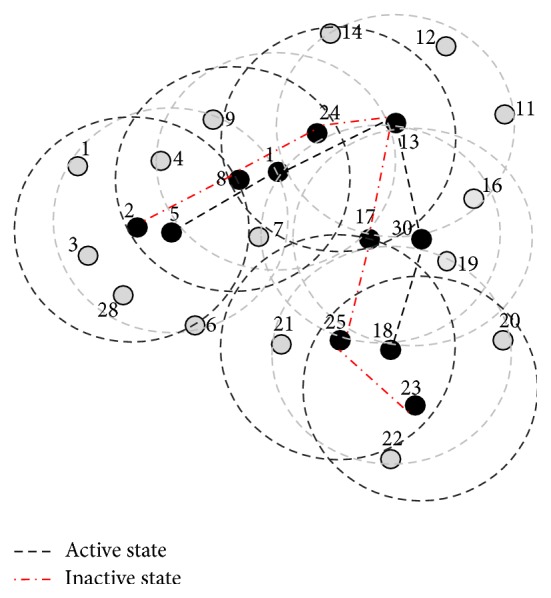
CDS with activity scheduling.

**Figure 6:**
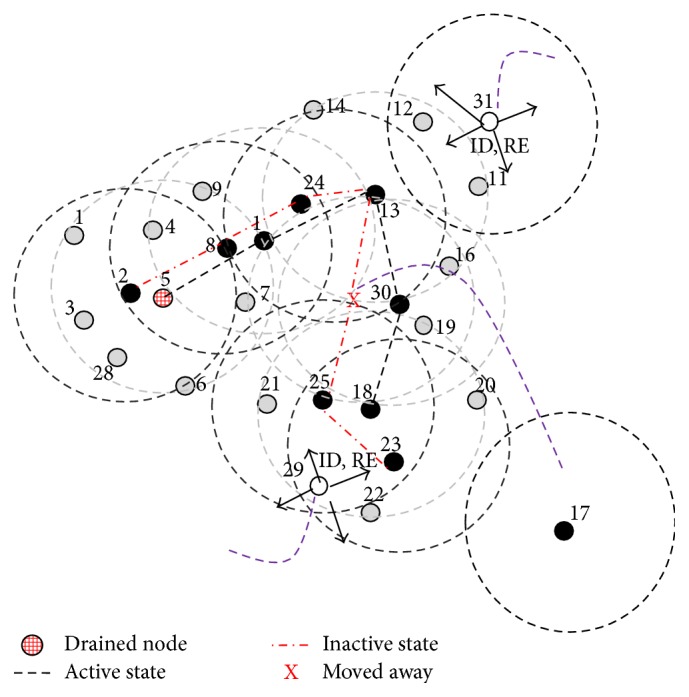
The energy efficient optimal CDS maintenance phase after Δ*T* time with new nodes 29 and 31. Moved-out node 17, drained-out node 5.

These phases are illustrated in Figures 4 and 5. According to the algorithm, all the dominators (BLACK) select the counterparts locally and perform activity scheduling. Here in the MANET node 5 selects node 2 as the counterpart and node 1 selects the union of node 8 and node 24 as counterparts, because their combined *N*_1_ list is equivalent to the neighbour list of node 1. Similarly node 17 and node 30 are counterparts. Node 18 selects union of node 25 and node 23 as the counterparts. Node 13 has no equivalent node or union of nodes with similar *N*_1_ list; in this case node 13 will not perform activity scheduling locally until an equivalent node/nodes join. When compared to other nodes node 13 drains out quickly. These cases are handled by maintenance phase. Activity scheduling is performed as shown in Figure 6. Nodes {(2,5), (1, (8,24)), (17,30), (18, (23,25))} perform activity scheduling locally. BLACK slashed lines (active connection) and RED slashed lines (inactive connection) indicate the scheduling. When compared to other algorithms the dominators will not run out of energy and lifetime of the network increases as shown in Figure 5.

*Algorithm Phase 5*. This phase is called maintenance/repair phase. Here the mobility of the node and the residual energy (RE) are considered for maintaining the optimal CDS. Here four cases are considered when a new node joins the network, existing node moves away, existing dominator moves away, and existing dominator runs out of energy. Every dominator executes this phase at frequent Δ*T* time intervals. A BLACK (dominator) node turns itself to RED when its energy becomes less than threshold TH; that is, it drains out (see Algorithm 5).

**Figure 7:**
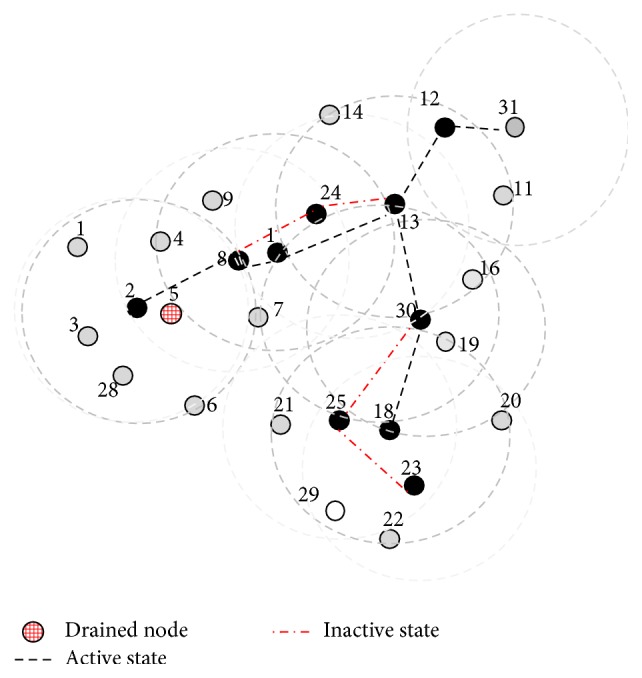
The final repaired CDS with minimal activity scheduling.

The above phase is illustrated in Figure 6. Here the dominator node 17 moved away so counterpart node 30 has become the BLACK node as shown in Figure 7. Node 5 has drained out and turned itself into RED and exchanged locally. So node 2 has become the dominator as shown in Figure 7. Two new nodes 29 and 31 have joined the network, where node 29 is nearer to the nodes 23 and 25. Node 31 joined network near nodes 11 and 12; now IDs are exchanged locally. Now node 12 will become the additional new dominator; as node 29 is already adjacent to two dominators, it turns itself into GREY as shown in Figure 7. The final optimal repaired CDS {2, (1, (8,24)), 13,12,30, (18, (23,25))} is shown in Figure 7. Here only the nodes {(1, (8,24)), (18, (23,25))} perform the activity scheduling.

## 5. Simulation Results

In this section, the simulation results of some energy efficient CDS algorithms are compared with our energy efficient optimal CDS algorithm with activity scheduling. We present simulations that illustrate the results of the algorithm and analyse the behaviour of our algorithm in various scenarios. We run simulations in ns-2 (version 2.34). The simulation parameters are listed in Table 2. Power considerations for the dominator node are given in Table 3. Here in promiscuous mode the dominator listens to its surroundings.

**Figure 8:**
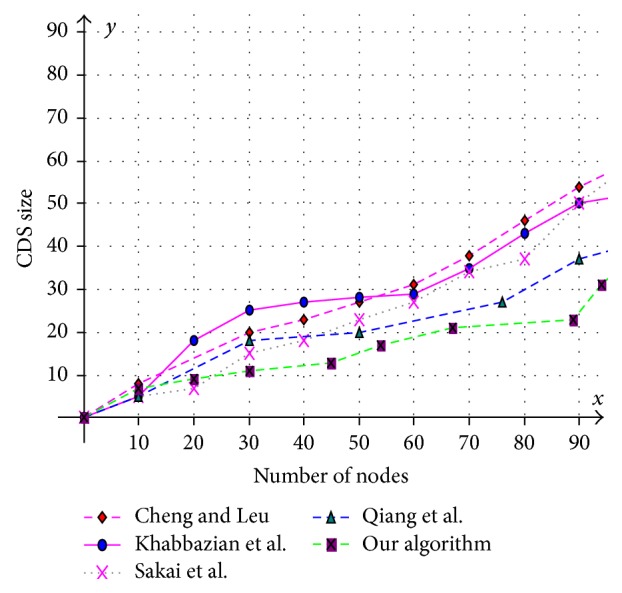
Number of nodes versus CDS size (transmission range = 25 mts and speed = 5 mts/s).

**Figure 9:**
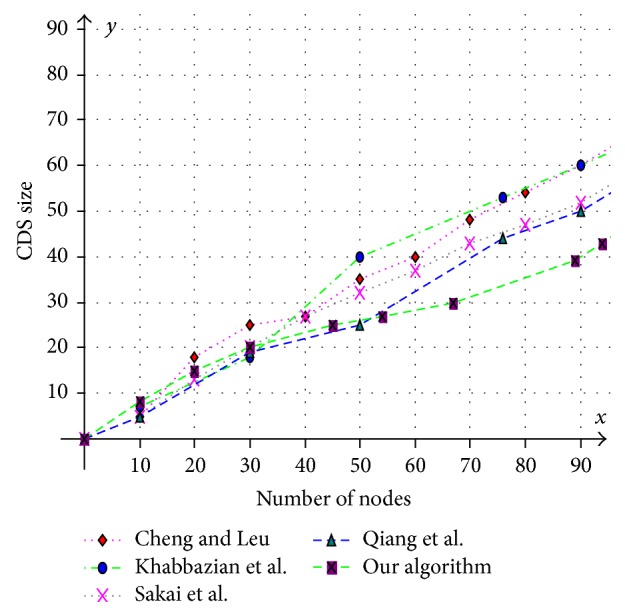
Number of nodes versus CDS size (transmission range = 25 mts and speed = 10 mts/s).

**Figure 10:**
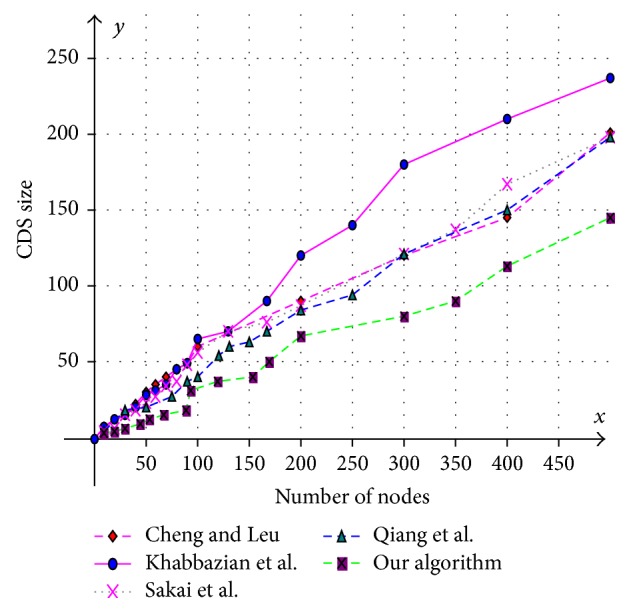
Number of nodes versus CDS size (transmission range = 50 mts and speed = 5 mts/s).

**Figure 11:**
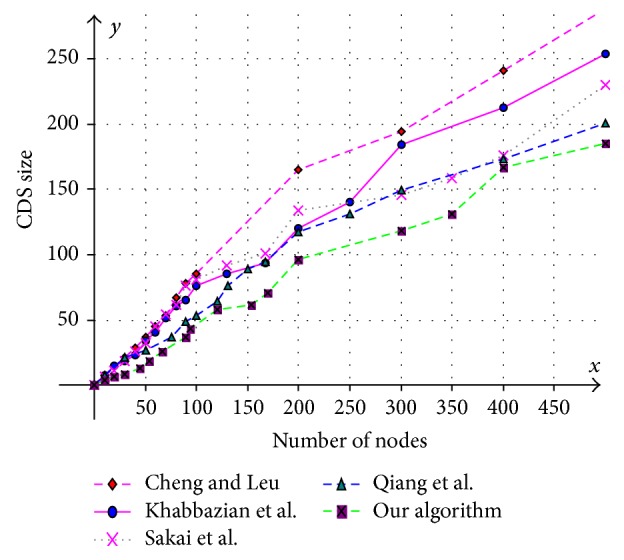
Number of nodes versus CDS size (transmission range = 50 mts and speed = 10 mts/s).

**Figure 12:**
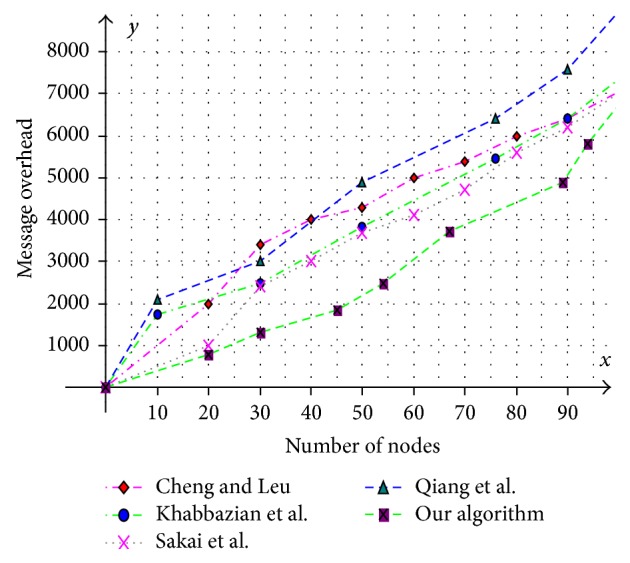
Number of nodes versus message overhead.

**Figure 13:**
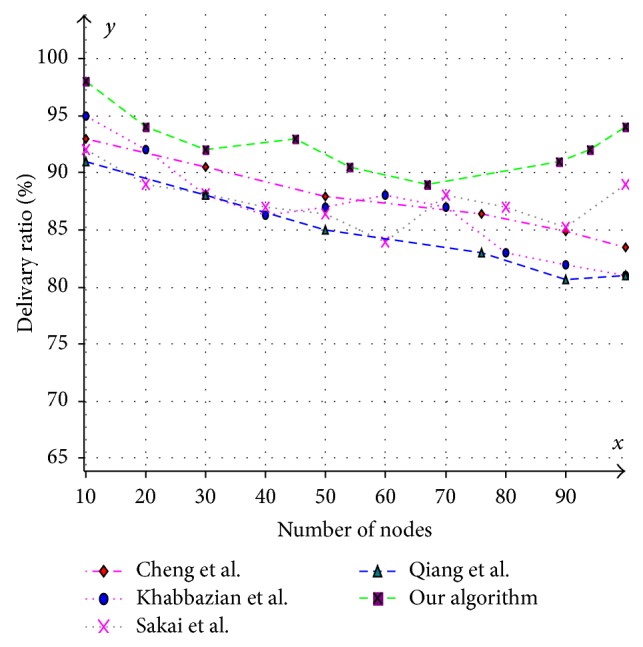
Number of nodes versus delivery ratio (%).

**Figure 14:**
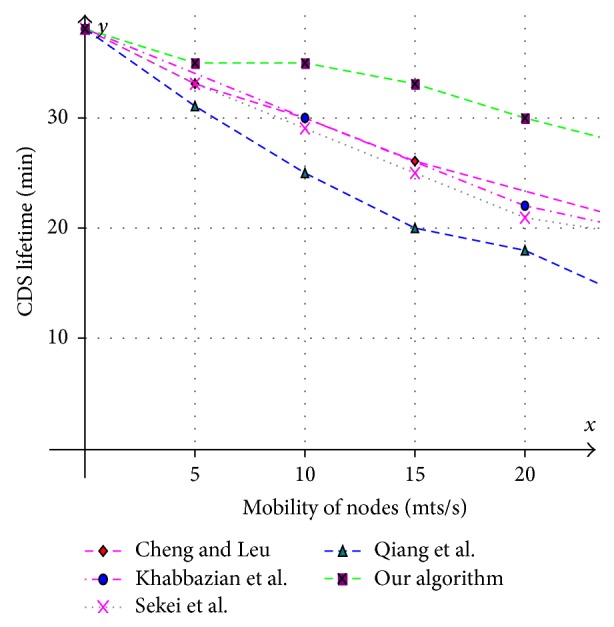
Mobility of nodes (mts/s) versus MCDS lifetime (min).

**Figure 15:**
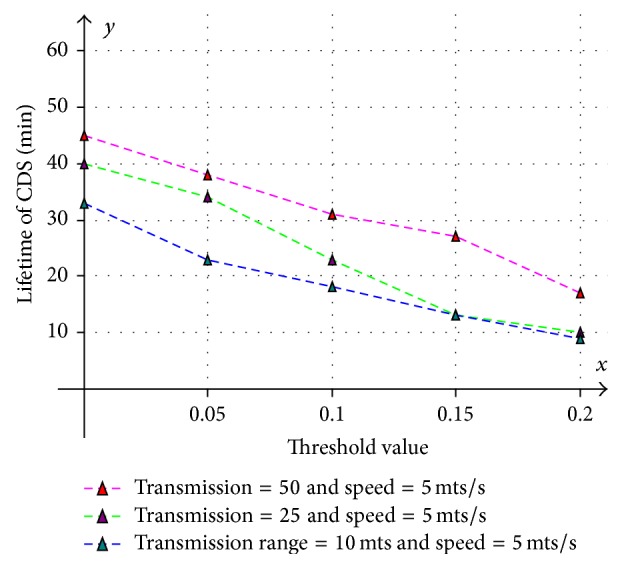
Lifetime of CDS (min) versus energy threshold values.

Various aspects like size of the CDS at various mobility rates, lifetime of the CDS at various mobility rates, and message overhead are observed and compared with existing CDS algorithms. Performance is analysed at sparse as well as dense networks. A sparse network is created with 100 nodes with transmission range of 25 mts at two different speeds 5 mts/s and 10 mts/s. Similarly a dense network is created with 450 nodes with transmission range of 50 mts at two different speeds 5 mts/s and 10 mts/s. A Random walk mobility model is used. The terrain area is about 1000 m × 1000 m.  Figure 8 shows the size of CDS versus number of nodes in sparse network with node's transmission range of 25 mts, at node speed of 5 mts/s.  Figure 9 shows the size of CDS versus number of nodes in sparse network with node's transmission range of 25 mts, at a node mobility of 10 mts/s. In both cases our algorithm performed well irrespective of speed and obtained optimal CDS size in sparse network when compared to the existing CDS algorithms.  Figure 10 shows the size of CDS versus number of nodes in dense network with node's transmission range of 50 mts, at a node mobility of 5 mts/s.  Figure 11 shows the size of CDS versus number of nodes in dense network with node's transmission range of 50 mts, at node mobility rate of 10 mts/s. In these cases also our algorithm performed well when compared to other algorithms because of its distributed nature.  Figure 12 shows the message overhead versus number of nodes with node's transmission range of 50 mts and speed 10 mts/s. Here our algorithm exchanged optimal number of messages when compared to the existing standard CDS algorithms, where the existing CDS algorithms have not performed activity scheduling, so the CDS repair phase message cost increased.  Figure 13 shows the delivery ratio versus number of nodes with transmission range of 25 mts and speed of 10 mts/s. Here also our algorithm performed well when compared to other algorithms. The graph in Figure 14 shows the lifetime of the optimal CDS versus mobility of nodes with node's transmission range of 50 mts. The lifetime of our algorithm is observed at different speeds of nodes 5 mts/s, 10 mts/s, 15 mts/s, and 20 mts/s. The lifetime of our algorithm in case of mobility is also good when compared to the existing standard CDS algorithms because of activity scheduling among neighbouring dominators in phases 3 and 4. So our algorithm is robust, reliable, and fault tolerant. The graph in Figure 15 shows the lifetime of the optimal CDS at various energy threshold values. Here the network is simulated with 200 nodes at various transmission ranges of 50 mts, 25 mts, and 10 mts and at a speed of 5 mts/s with threshold values of 0.05, 0.1, 0.15, and 0.2. The value of threshold at 0.1 gives better results and maintains the tradeoff between network lifetime and message cost.

## 6. Conclusion and Future Work

The major advantage of using activity scheduling algorithm is fast convergence and longer life even if CDS nodes move out due to mobility or energy drain. Here the energy of the dominators will not drain out due to activity scheduling. Only the dominator nodes are involved in further communication to establish the backbone and in maintenance of optimal CDS. In future work we will extend this to directed graphs and nodes with directional antennas.

## Figures and Tables

**Algorithm 1:**
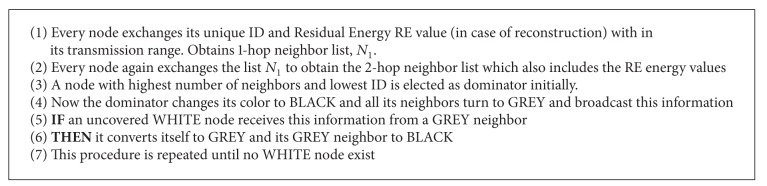


**Algorithm 2:**
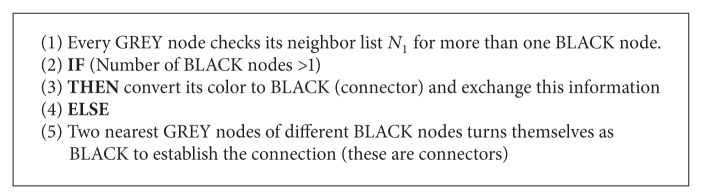


**Algorithm 3:**
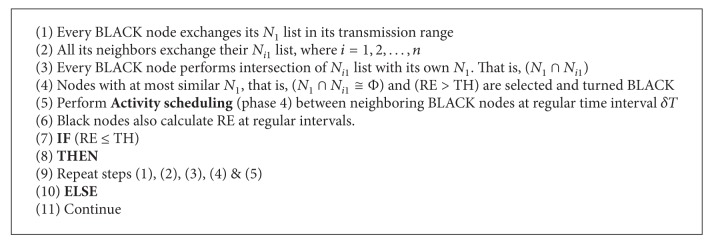


**Algorithm 4:**
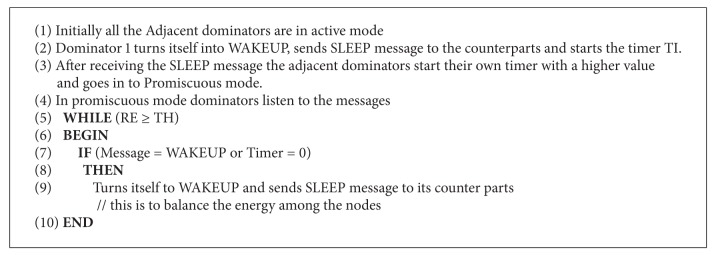


**Algorithm 5:**
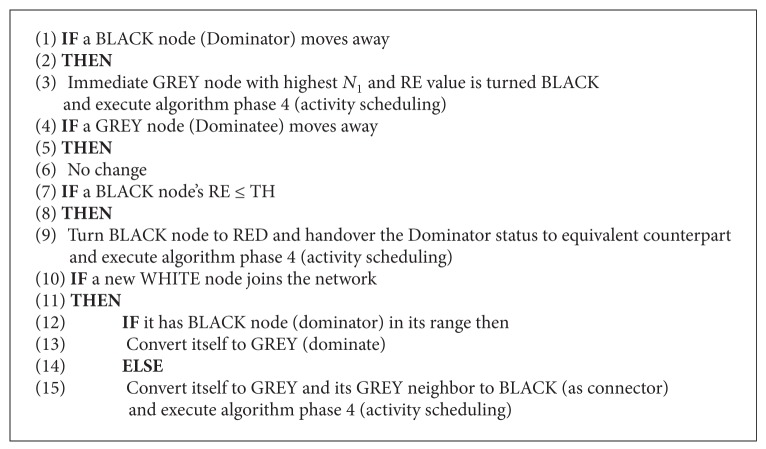


**Table 1:** Complexities of various CDS algorithms.

CDS algorithm	Classification	Time complexity	Message complexity
Guha & Khuller	Centralized	*O*(*n* ^2^)	*O*(*n* ^2^)
Cheng et al.	Centralized	*O*(*n*)	*O*(*n*)
Butenko et al.	Centralized	*O*(|*V*| · |*E*|)	*O*(*n* ^2^log^3^⁡*n*)
Alzoubi et al.	Distributed	*O*(*n*)	*O*(*n*log⁡(*n*))
Wu & Li	Distributed	*θ*(*m*)	*O*(Δ^3^)
Das et al.	Distributed	*O*(*n* ^2^)	*O*(*n* ^2^)
Stojmenovic et al.	Distributed	Ω(*m*)	*O*(*n* ^2^)
Dai et al.	Distributed	*O*(Δ^2^)	*O*(Δ)

**Table 2:** Simulation parameters.

Simulator	Ns2 (version 2.34)
Simulation area	1000 × 1000 m
Propagation	Two-ray ground reflection
MAC protocol	IEEE 802.11
Bandwidth	2 Mbps
Traffic	CBR
Transmission range	25~100 meters
Number of nodes	100~450
Maximum speed	5~25 m/sec
Mobility model	Random walk
Broadcast sessions	35
Broadcast rate	0.5–5 pkts/s
Message size	128 bytes
Hello interval	2 sec
Simulation time	60~100 minutes
Number of trials	75

**Table 3:** Power consumption table.

Parameter (node)	Power consumption (Watts)
Transmission	1000 mW
Receiving	500 mW
Promiscuous mode	100 mW
